# School-Based Participatory Response for Reopening During the COVID-19 Pandemic: A Case Study of a Metropolitan High School Implementing the Health Promoting School

**DOI:** 10.3389/fpubh.2021.578200

**Published:** 2021-04-06

**Authors:** Insook Kwon, Sunjoo Kang, Jin Sun Kim

**Affiliations:** ^1^Ewha Womans University High School, Seoul, South Korea; ^2^Department of Global Health, Graduate School of Public Health, Yonsei University, Seoul, South Korea; ^3^Department of Nursing, Chosun University, Gwangju, South Korea

**Keywords:** school disinfection strategy, safe environment, safety belt, health promoting school, school reopening

## Abstract

**Purpose:** This study aimed to analyze how a private high school in Seoul developed and executed a “school disinfection strategy” to ensure the students' right to study in a safe environment, and also to analyze the lessons learned from this process.

**Methods:** This was a case study of school health in a community-based school reopening during the COVID-19 pandemic. The study target was a 64-year-old private high school with 12 classes for each grade with a total of 1,100 students.

**Results:** A “school disinfection strategy” was set up at individual and class environment levels to protect students from the risk of infection. In addition, school health activities were carried out with a “personal protection safety belt” and “community protection safety belt” for effective implementation. To ensure a safe educational environment for high school students and to ensure smooth execution of face-to-face classes (in-person teaching), the “prevention safety belt strategy” was introduced in accordance with governmental guidelines to sequentially implement various preventive measures necessary to guarantee environmental safety of schools. Activating personal prevention safety belts by checking the symptoms of students when entering the school and during each class, and providing self-made disinfectants by spraying alcohol on wet-wipes were cost-effective and sustainable methods used in this school to prevent the spread of infection.

**Conclusions:** The experience of developing a prevention safety belt strategy to adapt the guidelines of the local education office to the school situation was presented. Focusing on the school community, as well as individual students and teachers, the concept of prevention safety belts helped to unite and stimulate voluntary participation of students in health promotion activities.

## Introduction

On January 30, 2020, the coronavirus disease 2019 (COVID-19) was declared as a Public Health Emergency of International Concern (PHEIC) by the Director General of the World Health Organization (1). Accordingly, the government of the Republic of Korea raised the country's Crisis Alert Level from “Attention” to “Serious.” Korea's Crisis Alert Level has four stages, wherein “Attention” refers to observing a new infectious disease outbreak overseas, and “Caution” refers to the outbreak entering the country whereupon the government implements measures for isolation to prevent the spread of infection. The first COVID-19 patient was detected in Korea on the day that PHEIC was declared. With the diagnosis of a second case, the national crisis warning level was elevated to “Alert,” and with the first COVID-19-related death on February 20 and a surge in cases to 433 on February 23, crisis warning was raised to the highest level (“Serious”). According to the Infectious Disease Control and Prevention Act of Korea, COVID-19 has been identified and categorized as a “Group 1 infectious disease—emerging infectious disease syndrome.”

The rapid increase in the number of infected patients was due to the improvement of the national infectious disease response system after the outbreak of the Middle East Respiratory Syndrome (MERS) in 2015. The system proactively checked and confirmed infected patients and effectively blocked further transmission through a tracking investigation (2). Meanwhile, due to the disturbance caused by the outbreak, the academic year which starts at the beginning of March for all educational institutes in Korea, was delayed for a week on February 22, 2020 when the crisis level was raised to “serious.” By the end of February, due to the increase in COVID-19 cases, the Ministry of Education postponed the start of school till March 23. However, with the constant rise of the public health crisis, school opening was postponed again, and schools finally opened on April 6.

School closure due to the COVID-19 situation can result in many serious consequences for students in several aspects. As a result of school closure, there was a serious concern that children from low-income families might not be able to receive free or subsidized lunches anymore, which could lead to an imbalance of nutrition and affect children's health, especially in the present circumstances when their families are unable to afford essential food items and other necessities due to the worsening economy (3). Furthermore, a group of experts recommended that prior to the reopening of schools, evidence of low infection rates in the community should be provided and systems to track new cases should be implemented to minimize the risk of infection among students (4).

With regard to school reopening, in the United Kingdom (UK), the Scientific Advisory Group for Emergencies (SAGE) reported seven “returning to school” scenarios, and warned that the push to reopen schools might lead to a new wave of infection (5).

Globally, community infection rates were considered as a critical determinant for the reopening of schools. In addition, the management of a safe school environment was considered as a prerequisite in the process of reopening, combined with a careful approach to minimize the risk of infection. In order to determine appropriate methods for achieving this goal, an indirect understanding of the actual practices in the school reopening process is essential. Therefore, a case study on a community-based school, displaying the experience of school-based participatory response after reopening can provide a suitable model for other schools in different countries during the current pandemic (6).

In Korea, school health includes “health promoting schools framework (HPS),” which was proposed by the WHO in the 1998 Ottawa Charter, to build a school environment that promotes healthy living and working. The HPS approach operates to promote children's health based on six key features: healthy school policies, social school environment, physical school environment, community links, individual health skills and action competencies, and health services (7). In Korea, an HPS pilot project was first carried out in 2009 in 16 Metropolitan and Provincial Offices of Education. Since 2012, the “Health Promotion School Model” has been included as a sub-project of educational innovation for creative school management, carried out by the Ministry of Education, Science, and Technology (8). In particular, links with the community and the formation of consensus among individuals were found to be important factors in the actual implementation of this model (9–12).

The purpose of this study was to analyze how a private high school in Seoul which conducted a health promotion school project, designed and executed a “school disinfection strategy” at a practice level to ensure the students' rights to study in a safe environment. It also aimed to analyze the lessons learned from the entire process. Thus, the current study provides information about necessary measures that need to be taken while preparing for school reopening and can help to reduce trial-and-error for the reopening process. It also contributes to the prevention of infection by guaranteeing students' right to study safely. The findings of this study can prove useful for schools in South Korea as well as other countries.

## Setting and Population

This was a case study on school health in a community-based school reopening during the COVID-19 pandemic. The study targeted a 64-year-old private high school in Seoul, with 12 classes for each grade level with a total of 1,100 students. This study was conducted with the approval of the principal of the target high school. The authors also requested exemption for the analysis of secondary data from the Institutional Review Board of Chosun University (IRB No. 2020-7-1-2) and received approval for the study from the same review board (IRB No. 2-1041055-AB-N-01-2020-33). This school had conducted a three-year pilot project on health promotion from 2012 to 2014, and even after conclusion of the project, teachers and students voluntarily performed various health promotion activities, such as health-related club activities and after-school health campaigns funded by the school's own budget.

## Situation and Strategies

### Early Stages of the COVID-19 Pandemic (January to Mid-May 2020)

Four weeks were spent to prepare the school's quarantine policy and COVID-19 manual version 1, since the end of January. After the initial orders for postponement of the new school semester during late February and early March, the Ministry of Education announced that classes will be conducted in an online format (13–17).

In addition, a school reopening preparation team was organized with the Vice-Minister of Education as its head, to manage disinfection and hygiene in schools, support learning, and prepare for reopening by consulting with the Metropolitan and Provincial Offices of Education (13). In order to improve the efficiency of student learning management, the Metropolitan and Provincial Offices of Education established online learning plans for each school, provided feedback for teachers on learning tasks, and established a system for individual online learning for students. This procedure differed for each provincial office. Since the period from the first to third week of March was a preparation period, each provincial office developed an online learning support team by expanding the infrastructure or by supporting teachers in their efforts to conduct online lessons. During the fourth to fifth week of March, before a complete distance learning mode was implemented, an online learning guide was made available and teachers' competency was improved with support from professionals or representative teachers of each school in the same district (Table 1). As a result, by the end of March, 2 million people were able to access the Korea Educational Broadcasting System's (EBS) online classes at the same time, and the Korea Education and Research Information Service (KERIS) strengthened 3,000 contents for the elementary and middle school students for the new semester. With the help of a network between the various governmental offices, 497 types of national and general equivalency diploma text books for elementary and middle schools were provided in the form of e-books. By selecting and operating a pilot school's distance learning program, a developmental model for generalized distance learning was established, and with a student information support project, computer and internet expenses for the students in low-income households were provided. This cooperation was possible due to an agreement signed on March 2, 2020 for the support of distance learning by the Ministry of Education, 17 Metropolitan and Provincial offices of Education, the KERIS, and the EBS, which attempted to establish a distance education operating model and reduce information gaps (16).

**Table 1:** Core strategic approach to online educational platform under COVID-19.

**Duration**	**Focus**	**Content**	**Technical support**	**Teacher assistance**	**School closure** **order (duration of** **closure)**
1st to 3rd week of March	Autonomous online learning support	E-learning platform, EBS contents: elementary 4,129, middle school 5,532, high school 18,859 e-text books	Expansion on infrastructure of major platform, preparing the expansion on the simultaneous access, Operation support for schools (3.10~), teachers (3.16~)	Operate teacher volunteer groups to support design of online learning and opening online classes	1st: 3.2~3.6 (1 week) 2nd: 3.9~3.20 (2 weeks)
4th to 5th week of March	Teachers managed online learning support	Community building through representative teachers and related organization participation	Distance learning using EBS	Online/distance learning guide, improving teachers' competency	3rd: 3.23~4.3 (2 weeks)
April	Opening of online school by stage	Third year of middle and high school: April 9~20	Same as the above	Same as the above	4th:4.6~4.8 (3 days)
		First and second year of middle and high school: April~ Fourth to sixth year of elementary school: April 16~	Same as the above	Same as the above	4th:4.6~4.15 (7 days)
		First to third year of elementary school: April 20~	Same as the above	Same as the above	4th: 4.6~4.17 (9 days)

*Reference: Ministry of Education (2020). Reorganized from the press release of February 23, March 5, March 17, March 26, March 31 (13–17)*.

However, as school opening was postponed four times and online learning became inevitable, a disinfection management team, student learning support team, and distance learning support team were added to this model (17).

### Strategic Operation for Safe School and Class Learning Amid the COVID-19 Pandemic

During the pandemic, teaching/learning could be provided through distance education (remote learning). However, face-to-face classes (in-person teaching) with safety measures to prevent infections were necessary for high school students in order to prepare them for University entrance examinations which were postponed to December, provided that the pandemic situation was alleviated.

**Figure 1:**
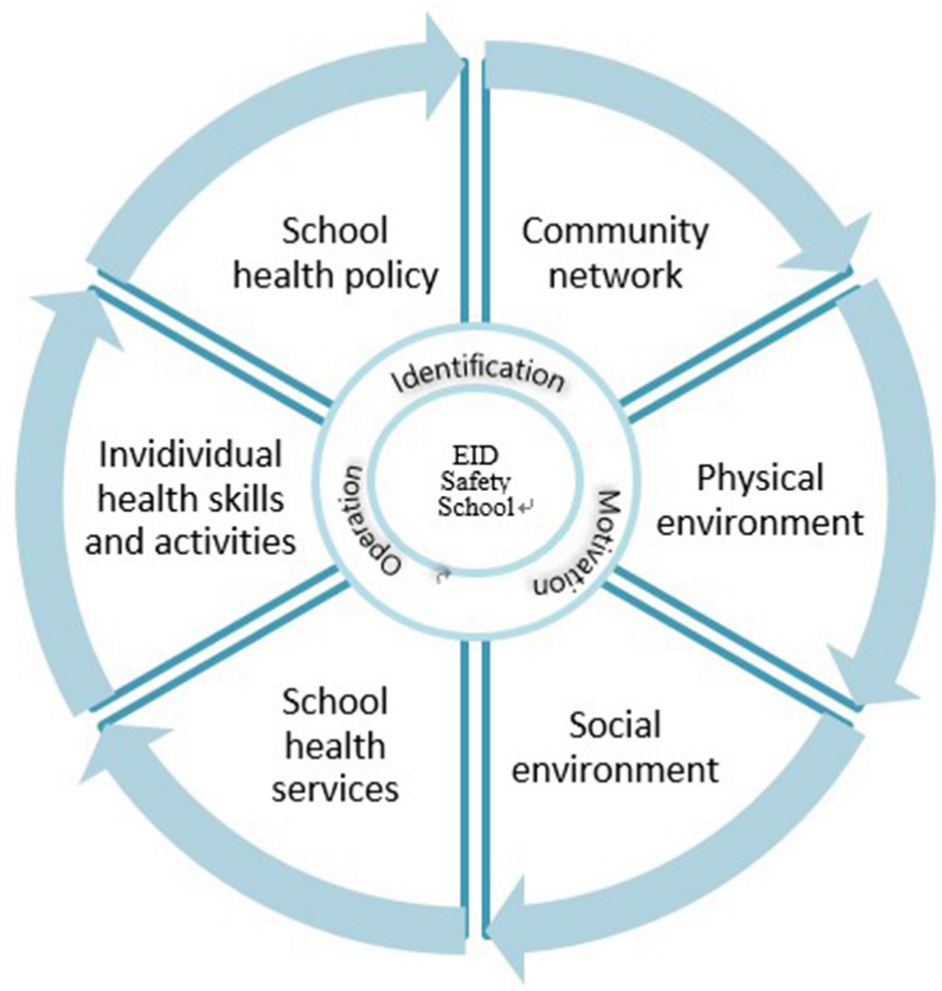
Personal and Community Prevention Safety Belt applied for the school health.

Therefore, in order to ensure a safe educational environment for high school students and ensure smooth execution of face-to-face classes, a “Prevention Safety Belt strategy” was introduced (Figure 1). This was a campaign encompassing personal level four prevention measures such as wearing masks, hand washing, cleaning desks, and maintaining a 2 m physical distance, as well as school environment regulations in accordance with governmental guidelines to minimize the risk of infection.

#### Operation of School Health Committee and Related Action Plans

Before the declaration of PHEIC by the WHO, the school health committee of the target high school discussed a response plan on how to implement safety measures by creating various scenarios. The school health committee is a standing committee, composed of the principal, vice-principal, and managing teachers for student affairs, school life, academic affairs, school administration, and school health; quarterly meetings are held by the committee to discuss key issues. Eight members of this committee met to form the draft of a manual guide on school disinfection strategy in response to COVID-19 every week in the first month, and thereafter, held periodic meetings every month till the present. For effective information sharing, they also used a social network service for group talks on their cellphones. After an offline or online meeting for students' school attending and leaving disinfection process, school environment disinfection and setting up desks and chairs with safe distances between them, and raising awareness and sharing information with parents, all of the tasks were discussed in detail in the cellphone group talk. The school's health teacher coordinated the committee's activities and the principal chaired the committee. The first activity was to establish the school's COVID-19 response plan in accordance with the guidelines of the Department of Education and the Center for Disease Control and Prevention. The committee set a goal to provide a safe learning environment in order to guarantee the students' right to attend school. A response plan was established and implemented for each element of the health promotion school model. In February and March, the main activities were a procurement plan for school quarantine items and planning online classes (Tables 2, 3). The school was able to procure the necessary number of masks, hand sanitizers, and environmental protection equipment with the support of the local government health authorities (18). By the end of April, two masks were provided to each student and teacher, and a certain number were stored in the school dispensary.

**Table 2:** Core strategic approaches to online education platform in the COVID-19 situation (2020).

	**January**	**February**	**March**	**April**	**May**	**June**
School Health policies	Activation of school health committee	Preparation of COVID-19 Response plan of EWHS	Planning for online class and teachers' competence building	Installation of thermal imaging camera and digital thermometer Quarantine	Identification of symptoms, monitoring and report	Identification of symptoms, monitoring and report
School health services	Agenda gathering for school health committee	Procurement plan for quarantine equipment	Procurement and distribution of quarantine equipment	Preparation of observation room, online education on self-management	Monitoring of school community and individual quarantine	Monitoring of suspicious symptoms and reporting cases to district health center
School environment			Securing environment protection products	Quarantine school environment	Distribution of two masks per person, daily quarantine after school	
Community networks			Refer for test on suspected symptoms		Supplement one day training for schoolteacher	
Individual skills and activities		Notification of delaying school opening: communication letter, SNS, school homepage	Guidance on online education	Online education: adherence to self-quarantine guidelines	Attending class: Wearing masks, hand hygiene, alcohol wipes for cellphone and desk	Student voluntary club for monitoring school community and individual quarantine safety belt
Parent care		Notification of delaying school opening	Notification of online education, self-quarantine of COVID- 19	Notification of online education and preparation for school attending	Guidance of class attendance and returning home upon identification of symptoms	

**Table 3:** Main contents of school prevention safety belt.

**Category**		**Detailed contents**
Individual prevention safety belt	Checking symptoms	Body temperature (fever), respiratory symptoms, nausea, loss of taste or smell, diarrhea, etc. Overseas travel history of self or family.
	Mask	All the time.
	Providing disinfection supplies	Disinfection products made by schools that contain alcohol in disposable wipes.
School community safety belt	Temporary observation room	Separated space with good ventilation.
	Thermal imaging camera	At the entrance of the school building.
	Disinfection by contracted company	All school facilities before and after school, in between periods, every day.
	Desk spacing in classroom	Keeping maximum distance between students, spacing front and rear as far as possible.
	Prevention supplies	Mask, thermometer, hand sanitizer, environment disinfectant, sterilized (alcohol) wipes.
Standards for attending school and returning home	Managing people in a household in isolation	Students and school staff must stay home if any member of their household is in isolation.
	Managing suspected case	Suspected patient with fever or respiratory symptoms is to be tested and treated at the medical institutions or screening clinic.
	Confirmed case	Students and all staff in self-isolation and convert to distance (remote) learning system. Additional actions to be taken in accordance to the epidemiological investigation.
	Standards for re- attending	(Isolated) When health center declares release. (Suspected symptom) When symptoms are relieved.
Monitoring		Monitoring by student voluntary club activity: Hand sanitizer location, usage, implementation of personal prevention safety belts.

Based on the committee's decisions, thermal imaging cameras were installed at the entrance of the school building, each classroom was equipped with a digital thermometer, and a space was allocated for a temporary observation room for students with suspicious symptoms, as shown in Table 2. The school's quarantine activities were notified to parents through various methods including letters sent to their homes, Social Network Service (Kakaotalk text message sending), and posts on the school's website homepage. Particularly, 1 week before school reopening, parents were guided to closely observe students at home and not send their children to school if any symptom was identified; these students were to visit the health center near their residential area for testing and notify the school regarding the same.

Through the online classes held in April, students were taught using educational videos produced by health teachers about emerging infectious disease prevention and self-management, as well as the importance of wearing masks, hand hygiene, and social distancing. From May 13, classes began for third year students in the high school, and subsequently for all other grades. Two weeks after the commencement of school, second year students who has been part of a voluntary club on health promotion requested permission to form a club and participate in personal and school quarantine activities. Led by a second-year student, the club had a total of seven members, including two first year students and five second year students. They got involved after the school authorities developed their own disinfection strategy and manual. Therefore, student club activities mainly supported students' compliance of the disinfection guide issued by the school, as well as a 15-min video on the prevention of Corona-blue, recorded by the students and mentored by the school health committee. This video was presented once to students of each grade, in mid-July, before the summer vacation.

#### School Community Prevention Safety Belt

Physical environment, social environment, and community network are the three elements of the health promotion school model which were important in the school community prevention safety belt to enable in-person teaching.

For physical environment prevention, first, an isolated observation room was required for those who exhibited symptoms, and this room needed to be apart from the regular school healthcare room. Therefore, the observation room was placed in a sunlit, well-ventilated space which was adjacent to the healthcare room but separated from the classrooms, and the teachers were notified to direct students with suspicious symptoms to the observation room immediately. Second, during the period of school attendance, all classrooms and other places used by students, such as the cafeteria, restrooms, and hallways, were sterilized by contractual external experts every day after the students were dismissed. In addition, the cleaning staff employed by the school frequently cleaned the entrance of the school building, the door handles of each classroom, and the bathrooms with environmental disinfectants after lunch, each new period, and before and after school hours. Third, thermal imaging cameras were installed at the entrance of the school building to measure body temperature before entering the building. Fourth, at least one digital thermometer was placed in every classroom. Fifth, the desks in the classrooms were rearranged to maintain a gap of at least 1 m or more (19). Since the number of students attending was reduced to only two-thirds of the total students, the school set a bi-weekly attendance policy for first and second year students, and no changes for third year students.

For social environmental prevention, in addition to compliance to the general governmental guidelines, parents and students were informed of the rules that they should adhere to at home. They were asked not to send the students to school if they exhibited any symptoms. In case any symptoms appeared during a class, the student was to be isolated in the observation room immediately and parents were to come and take the child to a nearby screening clinic. Community network has been a catalyst in promoting this prevention safety belt. From mid-January, the guidelines and materials issued on COVID-19 from the Ministry of Education were updated daily, and in order to respond effectively to school health prevention, the Severance Disaster Medical Education Center and the Seodaemun Public Health Center cooperated to provide one-day training on infection management that health teachers/school nurses required in order to prepare for school opening. The training consisted of basic lectures on COVID-19, wearing and changing practice for personal protective equipment level D, response procedures under suspected and confirmed cases of COVID-19, and questions and answers with infectious medicine experts.

This training was requested by the first-line teachers from the hospital's infection control room, and the authorities understood the seriousness of the situation and responded promptly by conducting the training within 2 days.

#### Personal Prevention Safety Belt

Individual health skills and abilities, school health services, and school policy are the three elements of health promotion school model which are important in personal prevention safety belt to enable face-to-face classes.

With regard to individual health skills and abilities, teachers and students were instructed to frequently clean their desks and mobile phones with alcohol-included wet wipes for at least 1 minute and also to frequently sterilize their hands with hand sanitizers located throughout the hallways. Before each class began, teachers conducted a health checkup on students, such as body temperature over 37.5°C and respiratory symptoms, and also confirmed whether the masks were properly worn. Above all, it was necessary to continuously monitor students' compliance to hygiene protocols (Table 3).

School health services included setting up a temporary observation room, placing necessary disinfectant supplies, taking actions based on standard algorithms upon receipt of students' symptom-related reports from the teacher, contacting parents of any student with fever, and so on. The status of symptomatic occurrence in school members was reported to the competent office of education through a computerized system, and it was possible to check the situation of nationwide confirmed cases and self-isolators in real time.

Regarding school health policy, a school health committee was convened, and training was provided to teachers as described in section School Community Prevention Safety Belt. This training was given first to teachers in charge of the third grade and subsequently to other teachers, since third year students were the first to restart face-to-face learning.

The contingency actions, such as directing persons with suspicious symptoms to go to the nearby screening clinic for appropriate tests and treatment, and if any confirmed case occurs, all students, teachers, and staff must self-isolate and classes should return to distance mode, were clearly documented. Furthermore, for convenient epidemiological investigations, all basic contacts and contact routes for symptomatic individuals were recommended to be recorded.

In addition, detailed guidelines on school health prevention were established, including the following specifics: the quarantine period included self-isolation for 14 days, and those under quarantine would be released if a confirmed negative report was obtained from the screening clinic upon retesting on the 13^th^ day of isolation. As for the suspected cases, they were to return to school only after all symptoms had disappeared.

#### Special Considerations for Sustainability

Though the school had a strong school disinfection strategy, one teacher tested positive for COVID-19, possibly due to the enforcement of the new governmental guidelines for routine distance in daily life; these guidelines are much less rigid than the strict social distancing guidelines and therefore, there is a greater chance of spread of infection in this scenario. The teacher attended a meeting in the last week of June and experienced some symptoms of COVID-19 after she attended a social meeting outside school on June 21. Four days later, on Thursday morning, she visited the nearby screening center and was confirmed positive for COVID-19 on Friday. This information was reported to the school health committee, and the school authorities decided to close the school and shared this information with students and parents. Classes were shifted to the online mode. Senior (third year) students who had attended classes of this teacher were classified as close contact persons and took the polymerase chain reaction (PCR) tests twice, once on the day when the teacher was confirmed as a positive case and next on the 13^th^ day of self-isolation to determine if they could be released from isolation. The other senior students were classified as “active monitoring” cases for 2 weeks; they were tested on the day when the positive case was confirmed, and were prohibited from visiting multi-use facilities as per the public guidelines. The school reopened for classes 1 week after the teacher had been confirmed positive. However, the senior students in quarantine did not attend. All of the 370 senior students attended school after 2 weeks, when they received PCR negative results. The other first and second year students' school attendance and learning were not affected.

Although training for school environment prevention and individual prevention safety belts for teachers and students was provided repeatedly, it was necessary to monitor actual compliance. A huge budget was needed to purchase and provide disinfectant-included wet wipes to the students as part of the individual prevention safety belt. As an affordable alternative, alcohol was added to plastic-tipped wet wipes (200 sheets) after consulting with community hospital infection control experts. Further, to monitor students' compliance to hygiene protocols, a group of second year students voluntarily formed a club and periodically monitored the consumption of hand sanitizers, inspected the students' individual prevention compliance status, and checked if the hand sanitizers were placed properly in the required places.

## Discussion

The strategic development of the prevention safety belts in this case study was not simply an implementation of the guidelines constantly issued constantly by the Ministry of Education and the Metropolitan Office of Education in the changing COVID-19 situation. To ensure the students' right to attend school in a safe school environment, members of the school staff participated actively in the formation of the school health committee. As a strategy for disease prevention, the school community prevention safety belt and personal prevention safety belt were promoted.

The school community prevention safety belt was developed in terms of physical and social environment, and personal prevention activities based on the six health promotion school indicators. In addition, formation of a student club to monitor whether major contents of the prevention safety belts were being followed at the individual level, had a positive effect on the fellow students; such an effect has also been shown in previous studies (10, 11). Activating personal prevention safety belts by checking the symptoms of students when entering the school and during each class, and providing self-made disinfection products were cost-effective and sustainable methods that can be applied easily in other schools. Meanwhile, the prevention safety belt activities led by the school health committee were possible because the teachers' capacity and understanding of school health improved due to the health promotion school experience. In addition, the students' efforts of forming a voluntary group to perform prevention safety activities, such as placing sanitizers in various areas around the school and monitoring the usage and moving flow, can also be applied as a strategy to encourage active participation of students in other schools during the current pandemic.

This study introduced the overall disinfection activities during the process of school reopening amid the COVID-19 pandemic in a high school located in the capital city of Korea, where the health promotion school model was established. In addition, the experience of developing a prevention safety belt strategy to adapt the guidelines of the local education office to the school situation was presented. Furthermore, under the new guidelines of routine distance in daily life, chances of being exposed to the pathogen are much higher. In this case, the school had one positive case; however, they overcame the challenges associated with it since they were well-prepared for such a situation. The teacher took a test as soon as she experienced mild symptoms, and all necessary precautions were taken to prevent the further spread of infection. Focusing on the school community, individual students and teachers, and the concept of prevention safety belts helped to unite and stimulate voluntary participation of the students, which is expected to contribute to the improvement of health maintenance by utilizing the health promotion school indicators under any circumstances.

## Limitations

This case study was conducted to share how a school was able to effectively respond to the public health emergency in the current times. It has some limitations of numerical representation and generalizability because we did not include various high school cases; we investigated only one school due to lack of time. However, we tried to balance various aspects to examine, describe, and suggest a practical strategy and action plan for a school disinfection strategy during an infectious disease outbreak.

## Data Availability Statement

The raw data supporting the conclusions of this article will be made available by the authors, without undue reservation.

## Ethics Statement

This study was conducted with the approval of the principal of the target high school. The authors also requested exemption for the analysis of secondary data from the Institutional Review Board of Chosun University (IRB No. 2020-7-1-2) and received approval for the study from the same review board (IRB No. 2-1041055- AB-N-01-2020-33). Written informed consent was not required for this study in accordance with national guidelines and local legislation because we only analyzed the committee's meeting minutes without any personal identifiable information.

## Author Contributions

IK was a major contributor in writing the manuscript, the conception and study design. SK and JSK contributed to critical revision of the manuscript. All authors read and approved the final manuscript.

## Conflict of Interest

The authors declare that the research was conducted in the absence of any commercial or financial relationships that could be construed as a potential conflict of interest.

## Abbreviations

PHEIC: public health emergency of international concern
MERS: middle east respiratory syndrome
UK: United Kingdom
SAGE: scientific advisory group for emergencies
HPS: health promoting school
EBS: Korea educational broadcasting system
KERIS: Korea education and research information service
KCDC: Korea centers for disease prevention and control (renamed as Korea Disease Control and Prevention Agency on September 12 2020).

## References

[1] World Health Organization. A joint statement on tourism and COVID 19-UNWTO and WHO call for responsibility and coordination (2020). Available online at: https://www.who.int/news-room/detail/27-02-2020-a-joint-statement-on-tourism-and-covid-19---unwto-and-who-call-for-responsibility-and-coordination (accessed June 15, 2020).

[2] Korea Centers for Disease Control and Prevention. The 2018 MERS response in the republic of Korea (2019). Available online at: http://www.cdc.go.kr/board.es?mid=a20504000000andbid=0014andact=viewandlist_no=365437andtag=andnPage=1 (accessed April 24, 2020).

[3] Rundle AG Park Y Herbstman JB Kinsey EW Wang YC. COVID 19 related school closings and risk of weight gain among children. Obesity. (2020) 28:1008–9. 10.1002/oby.22813 32227671 PMC7440663

[4] Wise J. COVID 19: delaying school reopening by two weeks would halve risks to children, says iSAGE. BMJ. (2020) 369:m2079. 10.1136/bmj.m2079 32444387

[5] Wise J. COVID 19: Push to reopen schools risks new wave of infections, says independent SAGE. BMJ. (2020) 369:m2161. 10.1136/bmj.m2161 32467094

[6] Smith ML Levkoff SE Ory MG. Community case study article type: criteria for submission and peer review. Front Public Health. (2016) 4:56. 10.3389/fpubh.2016.00056 27148510 PMC4830820

[7] World Health Organization. Health-promoting schools series 5; Regional guidelines development of health-promoting schools, - A., framework for action (1996). Available online at: https://apps.who.int/iris/handle/10665/206847 (accessed June 15, 2020).

[8] Korea Health Promotion Institute. Operating Manual for Health Promotion School. Seoul: Korea Health Promotion Institute (2014).

[9] Kim MJ. A systematic reviews on the effectiveness of foreign health promoting school. J Korean Soc Sch Health. (2014) 27:169–80. 10.15434/kssh.2014.27.3.169

[10] Ayi I Nonaka D Adjovu JK Hanafusa S Jimba M Bosompem KM. School-based participatory health education for Malaria control in Ghana: engaging children as health messengers. Malar J. (2010) 9:98. 10.1186/1475-2875-9-98 20398416 PMC2865503

[11] Aranda K Coleman L Sherriff NS Cocking C Zeeman L Cunningham L. Listening for commissioning: a participatory study exploring young people's experiences, view, and preferences of school-based sexual health and school nursing. J Clin Nurs. (2018) 17:375–85. 10.1111/jocn.13936 28639330

[12] Kim MJ Kim SH. Analysis of health promoting schools: focusing on high schools. J Korean Soc Sch Commun Health Educ. (2018) 19:95–108. 10.35133/kssche.20181231.08

[13] Ministry of Education. Decision of opening of new semester for all kindergarten, elementary, middle, and high schools nationwide and preparation of supplementary strategy to protect and manage entering of Chinese exchange students. February 23. (2020).

[14] Ministry of Education. Announcement on the school opening of kindergarten, elementary, middle, high, and special schools. April 5, 2020. (2020).

[15] Ministry of Education. Decision of additional 2 weeks delay on school start of all educational levels nationwide. March 17, 2020. (2020).

[16] Ministry of Education. Preparation on the distance learning to minimize the learning gaps due to school closure. March 26, 2020. (2020).

[17] Ministry of Education. Primary online school start for elementary, middle, and high schools – Final version. March 31, 2020. (2020).

[18] Ministry of Education. Stocking the small face masks in the schools as prevention for the COVID 19 – Final version. March 30, 2020. (2020).

[19] Ministry of Health and Welfare. 15 days strong social distancing – Government leads the distancing practice. March 22, 2020. (2020).

